# Impact of Illegible Prescriptions on Dispensing Practice: A Pilot Study of South African Pharmacy Personnel

**DOI:** 10.3390/pharmacy10050132

**Published:** 2022-10-12

**Authors:** Tasneem Modi, Ntandoyenkosi Khumalo, Rubina Shaikh, Zelna Booth, Stephanie Leigh-de Rapper, Gillian Dumsile Mahumane

**Affiliations:** Department of Pharmacy and Pharmacology, Faculty of Health Sciences, School of Therapeutic Science, University of the Witwatersrand, 7 York Road, Parktown, Johannesburg 2193, South Africa

**Keywords:** illegible prescription, dispensing, pharmacist, pharmacy personnel

## Abstract

Illegible prescriptions are an illegal, frequent, and longstanding problem for pharmacy personnel engaged in dispensing. These contribute to patient safety issues and negatively impact safe dispensing in pharmaceutical delivery. To date, little is documented on measures taken to assess the negative impact posed by illegible prescriptions on South African pharmacy dispensing personnel. Therefore, this pilot study was performed to evaluate the ability of pharmacy personnel to read and interpret illegible prescriptions correctly; and to report on their perceived challenges, views and concerns when presented with an illegible prescription to dispense. A cross-sectional, three-tiered self-administered survey was conducted among pharmacy personnel. A total of 885 measurements were recorded. The ability to read an illegible prescription is not an indicator of competency, as all (100%) participants (novice and experienced) made errors and experienced difficulty evaluating and deciphering the illegible prescription. The medication names and dosages were correctly identified by only 20% and 18% of all participants. The use of digital prescriptions was indicated by 70% of the participants as a probable solution to the problem. Overall, improving the quality of written prescriptions and instructions can potentially assist dispensing pharmacy personnel in reducing illegible prescription-related patient safety issues and dispensing errors.

## 1. Introduction

A South African pharmacist is a healthcare professional registered in terms of the Pharmacy Act No. 53 of 1974, specializing in the knowledge of drug-related products [1,2]. Pharmacists are responsible for providing pharmaceutical care by exercising their roles as custodians of medicine [3]. Thus, one of the functions a pharmacist plays in this regard is the dispensing of medicine or scheduled substances on the prescription of an authorized prescriber (a person registered under the Health Professions Act, 1974 and Medicines and Related Substances Act 101 of 1965). This dispensing process comprises three phases, namely: (1) the interpretation and evaluation of the prescription; (2) the preparation and labeling of the prescribed medicine and (3) the provision of information and instructions to the patient to ensure the safe and effective use of medicine [3,4]. Phase I of the dispensing cycle is, in part, heavily reliant on the ability of the pharmacist to read the prescription instructions presented accurately.

A prescription is an order or instruction written or transmitted electronically by a medical practitioner that authorizes a specified patient to receive medicine from a dispenser [5]. A prescription needs to be legible to ensure that the patient receives the correct medication at the correct dosage to benefit from the prescribed therapy. The essential requirement is that the prescription be legible and clearly indicate all written details, instructions, and items to be dispensed. The legibility of a prescription is further governed by law; South Africa is guided by the World Health Organization’s (WHO) ‘Guide to Good Prescribing’ and the National Department of Health’s ‘prescription writing’ guidelines, and the ‘General Regulations of the Medicines and Related Substances Act 101 of 1965 (Regulation 33) [6,7,8]. Therefore, prescribers must comply with the requirements set out by the Medicines and Related Substances Control Act (1965). Unfortunately, in practice, South African pharmacists are regularly presented with and face the dilemma of deciphering what is referred to in this study as an “illegible prescription” [9,10]. Thus, an “illegible prescription” in this study refers to a poorly written prescription that risks being unreadable and thus increases the chance of the prescription being misread and a product incorrectly dispensed.

To date, limited studies have evaluated the impact of illegible prescriptions on the dispensers, particularly the pharmacy personnel. One South African study at the district hospital in Bloemfontein evaluated the legibility of the doctors’ handwritten prescriptions by comparing and assessing the accuracy of reading the prescriptions between the doctors, nurses and pharmacists [9]. According to the study, pharmacists read the prescriptions more poorly than doctors and nurses, which raises a concern as they (pharmacists) are the conventional medication dispensers. The study speculated that the observation may have been due to the fact that the pharmacy personnel were mainly doing their community service training and had not been working with the doctors for long periods [9]. This speculation introduces and suggests the role of exposure and familiarity with certain prescribers’ handwriting as factors influencing the ability to read an illegible prescription.

Illegible prescriptions could result in several adverse outcomes for pharmaceutical service delivery. The inaccessible prescription instruction may negatively impact the pharmacists’ ability to efficiently carry out their function as a dispenser, by making the initial activity of reading in Phase I of the dispensing cycle challenging. Further outcomes include the increased probabilities of inaccurate interpretation of prescriptions, delays in pharmaceutical service and lethal patient outcomes [9,11,12].

As designated dispensers and custodians of medicine, pharmacy personnel are uniquely placed to highlight the negative impact of illegible prescriptions and the associated challenges concerning delivering quality pharmaceutical care. Therefore, this small-scale preliminary study was performed to evaluate pharmacy personnel’s ability to read illegible prescriptions and report on their perceived challenges, views and concerns when presented with an illegible prescription to dispense. This small-scale preliminary study was conceived in an effort to bring awareness and provide evidence for the challenges impacting pharmacy personnel in performing their duties in pharmaceutical service delivery while re-emphasizing the importance of legibility of prescriptions and urging offending prescribers to take note of the potential complicit act of maleficence that illegible prescriptions propagate for the patient.

## 2. Study Aims and Objectives

This study aimed to determine the impact of illegible prescriptions on dispensing practice among South African pharmacy personnel. Two overarching study objectives addressed the aim as mentioned: (1) assessing the pharmacy personnel’s ability to evaluate an illegible prescription accurately; (2) reporting on the pharmacy personnel’s perceived concerns, views and challenges encountered that are associated with dispensing an illegible prescription.

## 3. Materials and Methods

### 3.1. Ethics Approval

Ethical clearance was granted by the respective tertiary academic institutions Human Research Ethics Committee (Medical) and assigned the ethics clearance number M210533. Participants were provided with the relevant participant information and gave their written informed consent for inclusion before participating in the study. The study was conducted in accordance with the Declaration of Helsinki, revised in 2013 [13].

### 3.2. Study Design

This small-scale preliminary cross-sectional study utilized a self-administered survey evaluating the impact of illegible prescriptions on dispensing practice among pharmacy personnel in the Gauteng province of South Africa. Prescriptions were obtained from a local community pharmacy in Johannesburg (Gauteng, South Africa). The five prescriptions were randomly selected based on their illegibility. Sampling followed a purposive, random sampling strategy, and the data were gathered through an anonymous, self-administered questionnaire. The online (via Google Forms) self-administered survey targeted pharmacy personnel involved in dispensing prescriptions.

### 3.3. Study Population and Sample Size

The study participants included South African Pharmacy Council (SAPC) registered pharmacy personnel practising in South Africa: pharmacists, pharmacist interns, pharmacists completing their community service, pharmacist assistants (qualified post-basic) and pharmacy students. As it relates to Phase I of the dispensing cycle in the context of this study, the scope of the practice of the personnel is described as follows: a pharmacy student who has successfully completed their second year of study, a pharmacist’s assistant (post-basic) and a pharmacist intern may read and prepare a prescription under the direct personal supervision of a pharmacist in a pharmacy [4]. Community service pharmacists and pharmacists are responsible for providing pharmaceutical care and being accountable for meeting patient-related needs, including but not limited to, interpreting and evaluating a prescription [4]. Therefore, the study extended to third-year and fourth-year Bachelor of Pharmacy undergraduate students enrolled in their Bachelor of Pharmacy degree, as they share the scope of practice of a pharmacist assistant (qualified post-basic) under the direct personal supervision of a pharmacist in a pharmacy.

### 3.4. Data collection Tool

#### 3.4.1. The Survey

The survey was designed using the errors in the prescription writing checklist from the dissertation titled “Prescribing Errors at an Academic Teaching Hospital in Johannesburg” [14]. The authors’ permission was obtained to use and expand on the validated survey for the context of this study. The survey was three-tiered (Supplementary Material). The tiers are described as follows:

- Part 1: Demographic Information
- Part 2: Accuracy of reading an illegible prescription. The online survey comprised five prescriptions with illegible handwriting, and from the responses obtained, it was determined how accurately participants were able to decipher the prescribers’ handwriting. The illegible prescriptions were obtained from a local community pharmacy, and the prescription instructions were verified with the respective prescribers. The survey entailed the interpretation of the prescription (Phase I of the dispensing process) by listing the medication(s) prescribed, date, abbreviations, quantity prescribed, dosage and dosage form, as these are mandatory and regulated by the Medicines and Related Substances Control Act (1965). Participants were allowed to select ‘unsure’ if they could not determine what was written.
- Part 3: Open-ended questions explored the challenges in dispensing an illegible prescription and concerns/views on its consequences.

##### Scoring System

Statements assessing the accuracy of reading an illegible prescription were answered with three possible responses “yes”, “no,” or “not applicable”. The closed-ended questions were answered using the 5-point Absolute Category Rating (ACR) scale, which includes any one of the following responses: 1 = Bad (impossible to read and identify medication); 2 = Poor (illegible, words unclear, and ambiguity exists); 3 = Fair (some words illegible, some words legible, meaning unclear); 4 = Good (most words legible); and 5 = Excellent (all words are clear). The ‘not applicable’ option was included for those participants that did not believe they had adequate knowledge/experience to give a subjective response to some questions.

#### 3.4.2. Survey Dissemination and Study Procedure

Following ethics approval to conduct the study, the survey was administered using Google Forms, from which a link to the survey was generated. The Faculty of Health Sciences facilitated the dissemination of the survey invite and link to the third-year and fourth-year Bachelor of Pharmacy students. The link to the survey was further shared on various professional social media platforms such as LinkedIn, Facebook groups for professionals, WhatsApp and Telegram groups for professionals, and Twitter accounts related to professional pharmacy bodies. The survey required approximately 20 min to 30 min to complete. All surveys were retrieved with no manner of identifying where the survey had been completed or by whom. This was done to ensure anonymity for the participants. At no point in the survey was it necessary to fill in any personal details. Due to the study’s voluntary nature, no follow-up requests were made to participants who gave no consent or chose to withdraw.

### 3.5. Data Analysis

Data items were coded and transcribed onto a Microsoft Excel spreadsheet. Data were stored on a password-protected computer and only made available to the researchers and supervisors. Qualitative and quantitative methods were used to analyze data. Descriptive analysis (frequencies and percentages) was performed for variables included in the study. The qualitative analytic method of thematic analysis described by Braun and Clarke (2006) was used when approaching the open-ended questions [15]. This method helped identify patterns/themes within the data set.

## 4. Results

### 4.1. Demographic Data

A total of 59 pharmacy personnel agreed to participate in this study. The detailed demographic data are presented in Table 1. The mean age of participants was between 18-25 years of age. More than half of the respondents were less than 30 years old (91%) and had less than ten years of experience (90%). The majority (66%) of the participant profile reflected personnel in the training stages, e.g., pharmacist interns (14%), third-year pharmacy students (15%) and fourth-year pharmacy students (37%) requiring pharmacist supervision when dispensing prescriptions. Participants were mainly distributed to the community and institutional pharmacy sectors. Some participants operate in more than one sector of practice (working in academia and then locum at community pharmacies).

### 4.2. Prescription Reading Errors

There were five prescriptions (Table 2), each analyzed by 59 participants, which gave 295 prescription readings in total. Each prescription contained between two and five items (15 in total); therefore, a total of 885 measurements occurred for the five prescriptions presented.

In Table 3, the combined scores of one and two were the highest, as rated by the participants, indicating that the prescriptions used in the survey were indeed illegible. When looking at the five illegible prescriptions used in our study, Prescriptions 3 and 5 were rated as more illegible than the other prescriptions. None of the prescriptions scored five. Thus, the prescriptions in our study (Table 2) were likely primarily illegible, meeting the criteria of an illegible prescription.

**Figure 1:**
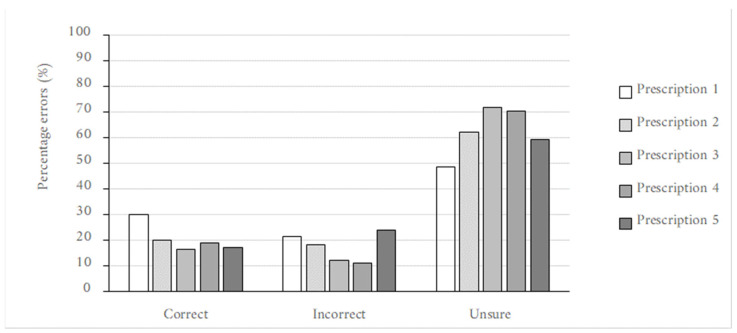
Column chart showing the varied abilities of pharmacy personnel to interpret the illegible prescriptions presented accurately.

Overall, a low level of accuracy in interpreting the prescription instructions was noted across all prescriptions by all pharmacy personnel, as shown in Figure 1. The incorrect and ‘unsure’ interpretations contributed to an alarmingly high percentage of the results. The combined incorrect and ‘unsure’ interpretations were 70%, 80%, 84%, 81% and 83% for Prescriptions 1, 2, 3, 4, and 5 respectively. In all instances, ‘unsure’ was the majority the response.

Table 4 represents the error percentages per prescription item by pharmacy personnel. Overall, mistakes occurred on all the prescriptions; no pharmacy personnel could read any of the five prescriptions without any mistakes. The majority of the pharmacy personnel were unsure of the dosage form (68%), medication names (62%), and abbreviations (58%). The highest percentage of incorrect answers were recorded for the quantity (41%) and dosage (23%). The date was the only prescription item interpreted most accurately by the pharmacy personnel (66%). Interestingly, the data in Table 4 indicate that ‘unsure’ responses were generally much higher than the correct and incorrect answers. Unfortunately, reading errors occurred with all the specific items checked on the prescriptions. In practice, this may lead to the incorrect dosing regimens and an incorrect drug being dispensed, further contributing to patient counselling informed by an inaccurate dosage regimen. This may negatively impact the patient–pharmacy personnel relationship as it relates to trusting them to provide safe and effective pharmaceutical care.

**Figure 2:**
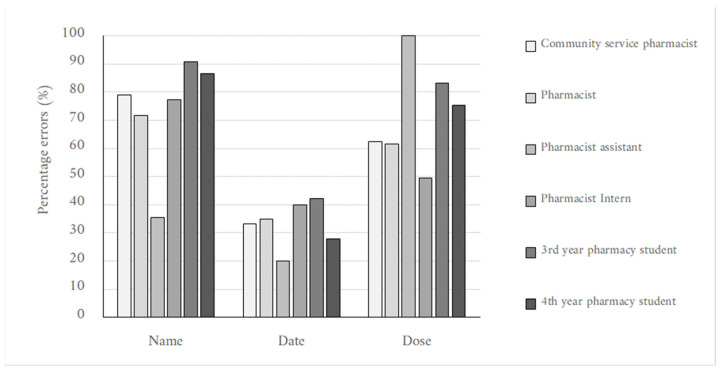
Column chart showing percentage errors made against the name, date, and dose by different pharmacy personnel groups across all prescriptions.

Figure 2 displays the percentage errors per item (name, date and dosage) for the respective pharmacy personnel groups. Only the name of the medications, dosage and date are displayed as some personnel omitted to write down their interpretation of the abbreviations, quantity and route of administration, despite it being presented as part of the prescription instructions. Overall, the pharmacists, community service pharmacists and pharmacist assistants read the prescriptions the best when compared to the other pharmacy personnel. Pharmacist interns followed this order, then fourth-year pharmacy students; the third-year pharmacy students were the least accurate of the group.

This study recorded some of the potential lawsuits and dispensing errors that could arise. From Prescription 1, Prexum^®^ (perindopril) was read as Diazepam and Lorazepam by 7% of pharmacy personnel. This would be an issue as the patient who requires an antihypertensive would receive a benzodiazepine, resulting in patient harm due to the adverse events of the wrong medication. An incorrect dosage of 4g for Prexum^®^ was read by 11% of the pharmacy personnel instead of the correct 4 mg prescribed. A high percentage of pharmacy personnel (45%) incorrectly answered that a quantity of “20” had been prescribed instead of “30”. In reference to Prescription 2, Prednisone was correctly answered by 15% of respondents, and a significant percentage of 71% of respondents answered they were unsure. It was noted that 10% of the pharmacy personnel answered Ridaq^®^ to this question. Ridaq^®^ is a diuretic used for hypertension or oedema; incorrectly dispensing this medication could result in unwanted reactions such as low blood pressure or adverse effects such as weakness, oliguria and postural hypotension. Combivent^®^ was also answered for Prescription 2. A significant percentage (64%) of respondents said they were unsure. This medication was correctly answered by only 10% of pharmacy personnel, and an even higher percentage of 12% incorrectly identified this item. The possible consequence of this error is that carvedilol is used for hypertension, and if Medicine 4 (prednisone) on the same prescription was also incorrectly prescribed as Ridaq^®^, this could result in medication interactions and lower blood pressure even further. Abnormally low blood pressure (hypotension) can cause dizziness and fainting and sometimes can be life-threatening.

### 4.3. Perceived Concerns and Challenges Encountered

All pharmacy personnel (100%), irrespective of years of experience and exposure to dispensing, confirmed that they experienced difficulty reading an illegible prescription (Table 5). Of these, 98% noted that they had asked a colleague for assistance when they had difficulty reading an illegible prescription. However, only 80% of Pharmacy personnel contacted the prescriber to confirm the medication/dosage if one is unsure, and 32% of pharmacy personnel hesitated when they made a call to the prescriber if there was difficulty reading a prescription; this is alarming given that all (100%) the participants reported facing difficulty in reading prescriptions. An alarming number of participants, 58%, confessed to making an error in interpreting or dispensing a prescription, further attributing it to ‘bad handwriting’.

The participants who selected “yes” to being hesitant to contact the prescriber in Table 5 were prompted to answer why they were hesitant. Three themes were highlighted: (1) pharmacy personnel did not want to be perceived as incompetent; (2) the prescriber is unavailable or difficult to get hold of via call; (3) upsetting the prescriber/the prescribers are rude and become agitated towards the pharmacy personnel when responding to queries regarding their handwriting, thereby upsetting the prescriber.

#### 4.3.1. How Pharmacy Personnel Deal with Illegible Prescriptions: Experiences

In the open-ended survey, we asked the question “Can you give a few words on the process you follow to decipher an illegible prescription”. The processes pharmacy personnel engage in when dealing with an illegible prescription were categorized under three themes: (1) contacting the prescriber, (2) asking a more seasoned colleague for assistance, and (3) checking in with the patient.

#### 4.3.2. Patient Frustration Due to Illegible Prescriptions

It was noted that 45 (76%) pharmacy personnel responded in agreement to the question, “Have you ever faced patient frustration as the delivery of service gets delayed when handed an illegible prescription?

### 4.4. Preferences of Pharmacy Personnel and Proposed Considerations

**Figure 3:**
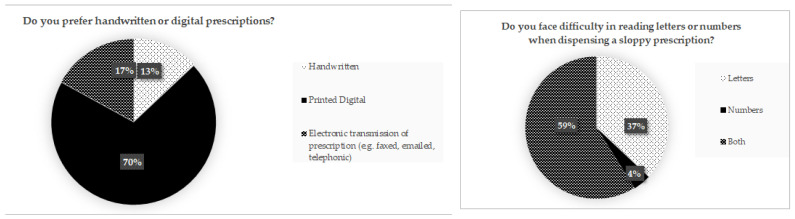
(**left**) Pie chart showing pharmacy personnel’s prescription preferences (*n* = 59) and (**right**) results of pharmacy personnel that have experienced difficulty reading letters/numbers (*n* = 59).

Most respondents preferred digital prescriptions (70%); electronically transmitted (handwritten prescriptions sent via electronic means (e.g., email, fax)) prescriptions were preferred by 17%, and handwritten prescriptions were preferred by only 13% (Figure 3). Most pharmacy personnel (70%) preferred printed digital prescriptions (Figure 3). A notable percentage of 37% of pharmacy personnel answered that they particularly faced difficulty reading letters compared to numbers. This is further supported by Table 4 and Figure 2, wherein the date (mostly number format in the prescriptions provided) had the least percentage error in interpretation across the pharmacy personnel.

## 5. Discussion

This study noted illegibility for all (100%) of the prescriptions. Prescription writing standards are prescribed and regulated by professional guidelines in South Africa [6,7,8]. The Medicines and Related Substances Act 101 of 1965 particularly states that every prescription for a medicine shall be written in legible print [16]. Despite the existence of professional standards and regulations thereof, illegible prescriptions persist.

Of note in this study is that the brand names were utilized instead of generic names for approximately 94% of the medications listed. This preference may result from brand names being generally more catchy and easier to remember as it relates to their indication and application (e.g., Flomist^®^ versus fluticasone propionate). Many studies have identified brand names contributing to prescription errors [9,17,18,19]. Brits and co-workers suggested that brand names are short, and many of them begin and end with similar letters, which may, in turn, cause confusion [9]. An example noted in our study was Allerway ^®^ being confused for Allergex^®^. It may be beneficial for medication to be prescribed by generic names to maintain uniformity [9,17]. This may further alleviate delays in the dispensing process where the dispenser may have to look up a new and unfamiliar brand name to try and identify the active component and ingredients.

An alarming number of participants (100%) self-reported making an error in interpreting or dispensing a prescription, further attributing it to ‘bad handwriting’. This is consistent with literature highlighting the association between illegible prescriptions and dispensing errors [8,20,21]. Some shared examples in this regard:

A pharmacist commented: “Dispensed Noxidem instead of Maxolon one time in 2018. The patient had a reaction and was hospitalized. The pharmacy council charged me and I had to pay a fine of around R11000 and also pay the patient for damages.”

A pharmacist intern commented: “I dispensed probiotics instead of antibiotics because I misread the *illegible* prescription. I have dealt with patient frustration on multiple occasions due to increased waiting times”.

Illegible prescriptions contribute negatively to the quality of pharmaceutical services in dispensing practice due to delays in the dispensing process, unintended dispensing errors and patient injury, and legal difficulties [22,23]. One such incident occurred when a prescription was written for “Amoxil tablets” (Amoxicillin), and the pharmacist misread this and dispensed “Daonil”, an anti-diabetic medication [8]. The patient did not have diabetes, resulting in permanent brain damage [8]. The court indicated that the prescriber owed a duty of care to a patient to write a prescription clearly and with sufficient legibility to allow for possible mistakes to be detected by a pharmacist. It was found that the prescriber had been negligent and not writing legibly resulting in a breach of his duty. The court concluded that the prescriber’s negligence had contributed to the negligence of the pharmacist. Another recent incident pertaining to the same medicine occurred in India when a patient suffered convulsions; further investigations revealed that the patient was prescribed an analgesic, “dudodil”, but was erroneously misread and given “daonil”, which caused her blood sugar levels to drop to the point she suffered from convulsions. This indicates the consequences of illegible handwritten prescriptions [24].

Illegible prescriptions presented difficulty in dispensing practices for all pharmacy personnel (novice and experienced) herein. In addition, an overall higher degree of illegibility compared to the literature was noted [9,25,26]. This may be attributed to most respondents being undergraduates (BPharm3 and BPharm4 students) in their training stages. At this early stage of their training, it is conceivable that they are likely less familiar with the drug names (generic and brand names) to apply familiarity towards deciphering what the words, symbols and letters could be. Seemingly, familiarity with the prescribers’ handwriting and long-term exposure may contribute to a slightly better ability to read an illegible prescription to some degree.

*A pharmacist assistant post-basic commented: “Identify other items to see if there is a pattern, i.e.,) pain script, antibiotic script. With an antibiotic script, you would expect to find a probiotic, cough mixture, antihistamine combination, nose spray and so on. Doctors also follow a prescribing style, so you get to know the doctors in the area and the combination of scripts they produce. So, they will always prescribe Augmentin with reuterina and flomist. So, you begin to recognize the patterns. That’s why unfamiliar doctor scripts are difficult to decipher.”*

A study at a South African hospital highlighted that pharmacists unexpectedly read the prescriptions worse than doctors and nurses. The observation was attributed to the fact that the pharmacy personnel were mainly doing community service and did not work with doctors for extended periods in the hospital, further citing that the doctors could read the prescription because they could recognize the handwriting and knew the medication prescribed [9]. Overall, the pharmacists, community service pharmacists and pharmacist assistants herein read the prescriptions relatively better than the interns and BPharm students. Pharmacist interns followed this order; after that, fourth-year pharmacy students and third-year pharmacy students were the least accurate of the group. This corresponds with the relative exposure to the pharmacy practice workplace. In practice, however, regardless of experience, pharmacy personnel not operating in an institutional pharmacy practice setting are less likely to develop long-term exposure and familiarity with a specific prescriber’s handwriting.

To eliminate the risks associated with illegible prescriptions, rules and regulations are provisioned for pharmacy personnel interventions [4,8,16]. Briefly, the prescribers need to be contacted to verify the instructions, and the pharmacy personnel are never to guess what the instructions are. The omission of and failure to take the necessary acts for patient well-being is deemed unethical or unprofessional conduct, subject to disciplinary steps by the Council under Chapter V of the Pharmacy Act 1974 [2,4]. This harkens to the professional judgement and ethical conduct of the pharmacy personnel as it pertains to the interest and well-being of the patient. Fortunately, a majority of personnel reported complying with contacting the prescriber for verification. However, approximately a third reported hesitancy towards the act. Sentiments in this regard within the different groups were:

*A pharmacist commented: “Yes. Patients start getting impatient that it’s taking ‘too long’. Also, patients lose faith in pharmacist abilities because it looks like we don’t know what we’re doing. Also, pharmacists get frustrated as well as often prescribers are not available to confirm, and patients must be sent back.”*

*A community service pharmacist commented: “Yes, because in the public sector doctors see a large number of patients so calling isn’t that efficient because he or she might have forgotten the patient and sending them is frustrating and time-consuming for the patient.”*

*A pharmacist intern commented: “In my opinion, it depends. If you get a bad prescription and you care about the patient’s health and well-being, you’ll call the prescriber to confirm. By doing this you’re offering a valuable service. Everything you do you should do it with quality, accuracy, and integrity. But then if you look at it from a time-consuming perspective, it does for sure affect the service delivery. That time you used to get hold of the doctor could’ve been time spent counselling the patient. You are probably wondering, “okay but you can counsel the patient afterwards”, yes well the queue in pharmacies gets pretty hectic and patients complain. But then again, those patients waiting in line doesn’t know the true value of this profession, which is not their fault. It’s 100% our fault. (I could go on and on and on about this one, but let me stop here)”.*

*A fourth-year pharmacy student commented: “Yes definitely, time-consuming. Could lead to mistrust from patient. Can lead to unnecessary tension with a doctor if you have to call them all the time, Doctors are often busy leading to more waiting and unhappy patients”.* The hesitancy to contact prescribers recorded by this study further causes concern as illegible prescriptions have been linked to errors in the past, and these errors can put the patients’ health at risk [9,24].

Written notes have been reported to take 46% longer to read than typed notes, according to a chart review comparing written and typed primary care progress notes [27]. The noted higher preference for digital prescriptions in this can be attributed to the envisioned potential benefits related to time, in combination with a lower probability of illegibility associated with e-prescriptions compared to handwritten prescriptions. Digital prescriptions eliminate prescription errors and time wasted on phone calls and call-backs to pharmacies; they thus may contribute to augmenting patient safety [21,28]. A respondent shared:

*A pharmacist intern commented: “it should start by the starting line: the doctor. So basically, have a mandatory typewritten prescription in place. I’m not an IT guru, but surely someone can develop some system in place to make things easier for both the doctor, nurses, and pharmacists. I am aware of the amount of patients doctors and nurses see a day, but surely there should be something out there”.*

### Limitations, Strengths and Interpretations of the Study

In reference to the interpretation of this work, several study limitations should be considered. The first is that the small-scale preliminary study results were obtained from 59 participants, whereas of 20 August 2022, the SAPC registered personnel statistics of pharmacists were reported at 16753, community service pharmacists at 859, pharmacist interns at 1340, assistants at 26031 and pharmacy students at 4115 [26]. The second is that the majority of the participant profile reflected personnel in the training stages (63%). It may be particularly insightful and worthwhile to pursue a more homogenous sample focused on pharmacists only. This is because registered responsible pharmacists ultimately supervise all the personnel mentioned herein; therefore, they may be able to provide an experienced perspective with a more encompassing interpretation of the impact of illegible prescriptions on dispensing practice (from the dispensing process, to patient interactions to personnel training) by virtue of their broader scope of responsibilities. It has been cited that “some pharmacists feel like kings after cracking an illegible prescription” [10]. Pharmacist-focused views on illegible prescriptions and their impact on dispensing practice may further aid in correcting misinformation and damaging sentiments by harkening to novice personnel (community service pharmacists, pharmacist assistants, pharmacist interns and students (BPharm3 and BPharm3)) that the ability to read an illegible prescription is not to be valued as a marker of competency. In the same breath, it was valuable for the study to bring in the experiences of BPharm3 and BPharm4 students as it relates to their training in the practice of the profession. Such groups should not go unchecked as the ability to read the letters, symbols and numbers on a prescription is a basic function that forms part of their training in their ability to carry out Phase I of the dispensing process under the direct supervision of a pharmacist. Their perspective may be of value in highlighting opportune areas of intervention and reinforcement for their educators and tutors in their education and training as it relates to reading and interpreting a prescription and the rules and regulations provisioned for pharmacy personnel interventions in this regard [4,8,16]. The third consideration is: that although anonymity was given to the participants, not all respondents provided written comments, and it is likely that those participants who provided comments had strong interests and opinions on this particular topic. Given a larger population size that is more reflective of the SAPC registered personnel population, the findings could be more generalizable. Thus, it is advised that the findings of this pilot study be used to draw attention to the challenge of illegible prescriptions in dispensing practice and not for making conclusions or generalizing to the entire population of the SAPC registered pharmacy personnel.

## 6. Conclusion and Study Recommendations

This small-scale preliminary study was able to show that illegible prescriptions are indeed an ongoing and longstanding problem for all pharmacy personnel sampled. Illegible prescriptions interfere with effective communication between the prescriber and dispensing personnel, increasing the chance of misreading prescription instructions and dispensing an incorrect product. The list of observed errors presented in this small-scale preliminary study highlighted some consequential and severe adverse drug reactions, which may ultimately compromise the pharmacy personnel-patient dynamic as it relates to trust in delivering quality pharmaceutical care. Overall, the majority of personnel struggle with both numbers and letters; therefore, efforts towards improved legibility with numbers and letters should be considered by prescribers going forward.

A preliminary recommendation is thus proposed for Health Science educators and training facilities (e.g., academic hospitals, places of internship and community service training) to further emphasize the importance of compliance to prescription requirements like legibility as part of their training. This may be achieved by embedding and harkening to the legal, practical and clinical implications of prescription illegibility in their respective learning activity designs. South African-specific monitoring, reporting and rehabilitation programs may be worthwhile to evaluate, develop, vet and implement for the purposes of bringing awareness, minimizing the frequency of illegible prescriptions and sharing practical methods of addressing concerns associated with illegible prescriptions. Conducting a study beyond this pilot study, with a larger SAPC personnel population, may be worthwhile towards more generalizable results to inform actionable solutions to the longstanding problem of illegible prescriptions.

## Figures and Tables

**Table 1:** Baseline demographic data of pharmacy personnel.

Demographic Characteristic	Category	Number of Participants (*n* = 59)	Frequency (%)
Age	18–25	42	71%
	26–30	12	20%
	31–39	3	5%
	>40	2	3%
Gender	Female	47	80%
	Male	12	20%
	Prefer not to say	-	0%
	Other	-	-
Pharmacy Personnel	Pharmacist	16	27%
	Pharmacist intern	8	14%
	Pharmacist assistant (qualified-post basic)	1	2%
	Community service pharmacist	3	5%
	Third-year pharmacy student	9	15%
	Fourth-year pharmacy student	22	37%
Current sector of practice	Retail/community	26	36%
	Locum (retail/community)	8	11%
	Hospital/Institutional	23	32%
	Academia	11	15%
	Industry/Manufacturing	3	4%
	Other	2	3%
Years of experience	<1 year	27	46%
	1–5 years	26	44%
	6–10 years	3	5%
	11–15 years	0	0%
	16–20 years	1	2%
	>20 years	2	3%

**Table 2:** Illegible prescriptions obtained from a community pharmacy.

Prescription	Correct Details
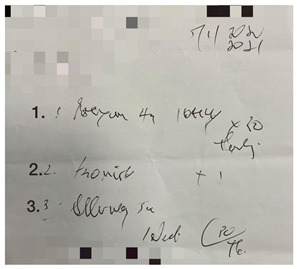	**Prescription 1:** Label 1: Prexum^®^ (Tablets) Label 2: Flomist^®^ Label 3: Allerway^®^ Date: 5 January 2021 Quantity medication 1: 30 Dosage medication 1: 4 mg
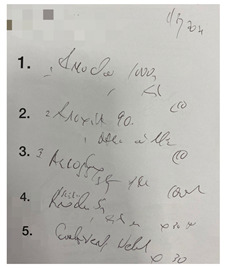	**Prescription 2:** Label 1: Amoksiklav^®^, (Bio- Amoksiklav) Label 2: Arcoxia^®^ Label 3: Alcophyllex^®^ Label 4: Prednisone Label 5: Combivent^®^ Nebuliser OR Duolin^®^ respules (generic) Date: 2 Ferbrary 2021 Label 4 quantity: 30 Label 2 dosage: 90 mg
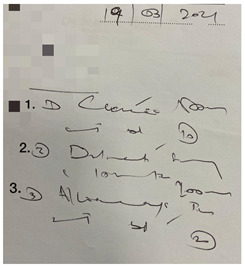	**Prescription 3:** Label 1: Clacee^®^ Label 2: Dilinct^®^ syrup Label 3: Allerway^®^ tab Date: 19 March 2021 Label 3 abbreviation: b.d. Label 3 quantity: 10 Label 1 dosage: 500 mg Label 2 dosage form: syrup (oral)
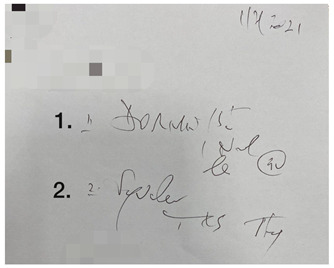	**Prescription 4:** Label 1: Dormicum^®^ Label 2: Synaleve^®^ Date: 1 Ferbrary 2021 Label 2 abbreviation: TDS, tds, Txd Label 1 quantity: 30 Label 1 dosage: 15 mg
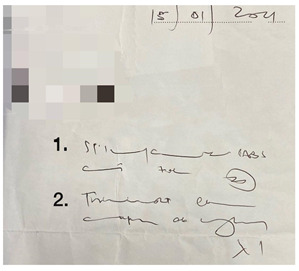	**Prescription 5:** Label 1: Stilpane^®^ Label 2: Travocort^®^ cream Date: 15 January 2021 Label 2 abbreviation: od Label 1 quantity: 20 Label 2 dosage form: Cream (skin) (dermal)

**Table 3:** The results of the legibility of the five prescriptions as rated by the participants.

How Legible Would You Rate the Prescription	Response									
	Participants (*n* = 59)									
	1		2		3		4		5	
	*n*	%	*n*	%	*n*	%	*n*	%	*n*	%
Prescription 1	41	69	14	24	3	5	1	2	0	0
Prescription 2	39	66	16	27	3	5	1	2	0	0
Prescription 3	47	80	10	17	2	3	0	0	0	0
Prescription 4	40	68	13	22	6	10	0	0	0	0
Prescription 5	50	85	8	14	1	2	0	0	0	0

**Table 4:** Summary of errors and correct answers for prescription items by all pharmacy personnel.

Prescription Item	Incorrect	Unsure	Correct
Name	13%	62%	20%
Date	23%	11%	66%
Quantity	41%	29%	30%
Dosage	23%	45%	32%
Abbreviation	5%	58%	37%
Dosage form	15%	68%	18%

**Table 5:** Frequency of illegible prescriptions and means of managing them.

Statements	Response					
	Total Participants (*n* = 59)					
	Yes		No		Not Applicable	
	*n*	%	*n*	%	*n*	%
Have you ever faced difficulty reading an illegible prescription?	59	100	0	0	0	0
Do you ask a colleague for assistance when you have difficulty reading an illegible prescription?	58	98	0	0	1	2
Do you consult WhatsApp/telegram/other social media groups for assistance when you have difficulty reading an illegible prescription?	16	27	39	66	4	7
Do you contact the prescriber to confirm the medication/dosage if ambiguity exists?	47	80	4	7	8	14
Are you hesitant to call the prescriber when you have difficulty reading a prescription?	19	32	34	58	6	10
Have you ever made an error in interpreting or dispensing a prescription due to bad handwriting?	34	58	25	42	0	0

## Data Availability

The data presented in this study are available upon request from the corresponding author. The data is not publicly available due to confidentiality obligations per the ethics guidelines of this study (ethics clearance certificate number M210553).

## Supplementary Materials

The following supporting information can be downloaded at: https://www.mdpi.com/article/10.3390/pharmacy10050132/s1, S1: A survey evaluating the pharmacy personnel’s ability to read an illegible prescription and the associated concerns and challenges in dispensing an illegible prescription.
