# Supplementary material for: Impact of Illegible Prescriptions on Dispensing Practice: A Pilot Study of South African Pharmacy Personnel
Source: Pharmacy (Basel). 2022 Oct 12;10(5):132. doi: 10.3390/pharmacy10050132 (PMC9609295; doi:10.3390/pharmacy10050132)
Supplement: Supplementary file 1 [file pharmacy-10-00132-s001.zip › pharmacy-1919978-supplementary.pdf]

S1: Online Survey

THE AIM OF THIS STUDY IS TO EVALUATE PHARMACY PERSONNEL'S READABILITY OF SLOPPY PRESCRIPTIONS AND WHAT THEIR CONCERNS OR CHALLENGES ARE IN DISPENSING SLOPPY PRESCRIPTIONS.

Demographic Questions:

Age:

- 18-25
- 26-30
- 31-39
- >40

Gender:

- Male
- Female
- Other

Pharmacy Personnel:

- Pharmacist
- Pharmacist Intern
- Pharmacist assistant (qualified-post basic)
- Community service Pharmacist
- Pharmacy Student 3rd year
- Pharmacy student 4th year

Highest Qualification:

Pharmacy sector:

- Retail/community
- Locum (retail/community)
- Hospital/institutional
- Industry
- Academia
- Other, Specify:

Years of experience:

- <1 year
- 1-5 years

- 6-10 years
- 11-15 years
- 16-20 years
- >20 years

Part 1: Survey assessing readability.

1. Have you ever faced difficulty reading a sloppy/illegible prescription?	○ Yes	○ No	
2. Do you ask a colleague for assistance when you have difficulty reading a sloppy prescription?	○ Yes	○ No	○ Not Applicable
3. Do you consult a WhatsApp/telegram/other social media groups for assistance when you have difficulty reading a sloppy prescription?	○ Yes	○ No	○ Not Applicable
4. Do you contact the prescriber to confirm the medication/dosage if ambiguity exists?	○ Yes	○ No	○ Not Applicable
5. Are you hesitant to call the prescriber when you have difficulty reading a prescription?	○ Yes	○ No	○ Not Applicable
6. Do you prefer handwritten prescriptions or digital prescriptions?	Hand written	Printed Digital	e-prescription (sent via e.g. gmail)

7. Do you face difficulty in reading the letters or numbers when dispensing a sloppy prescription?	Letters	Numbers	Both
8. Have you ever made an error in interpreting or dispensing of a prescription due to bad handwriting?	○ Yes	○ No	○ Don’t Know
Please answer the questions to follow in reference to the following attached prescription below<br>***Attached Sloppy Prescription Images***<br>(Note: This will be done for the following 5 different sloppy prescriptions with the same questions asked for each of them.)			

Prescription 1:

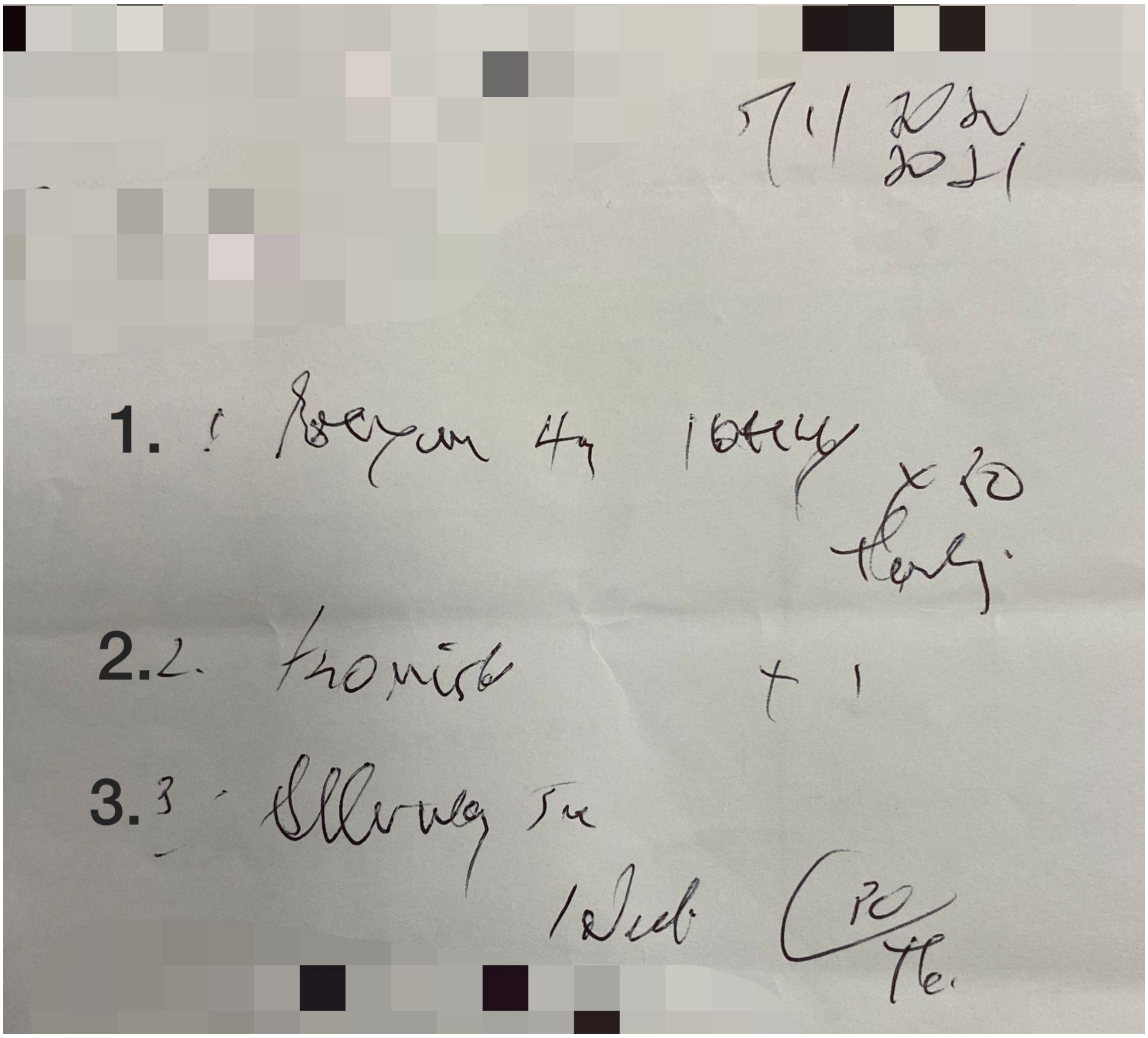

Prescription 2:

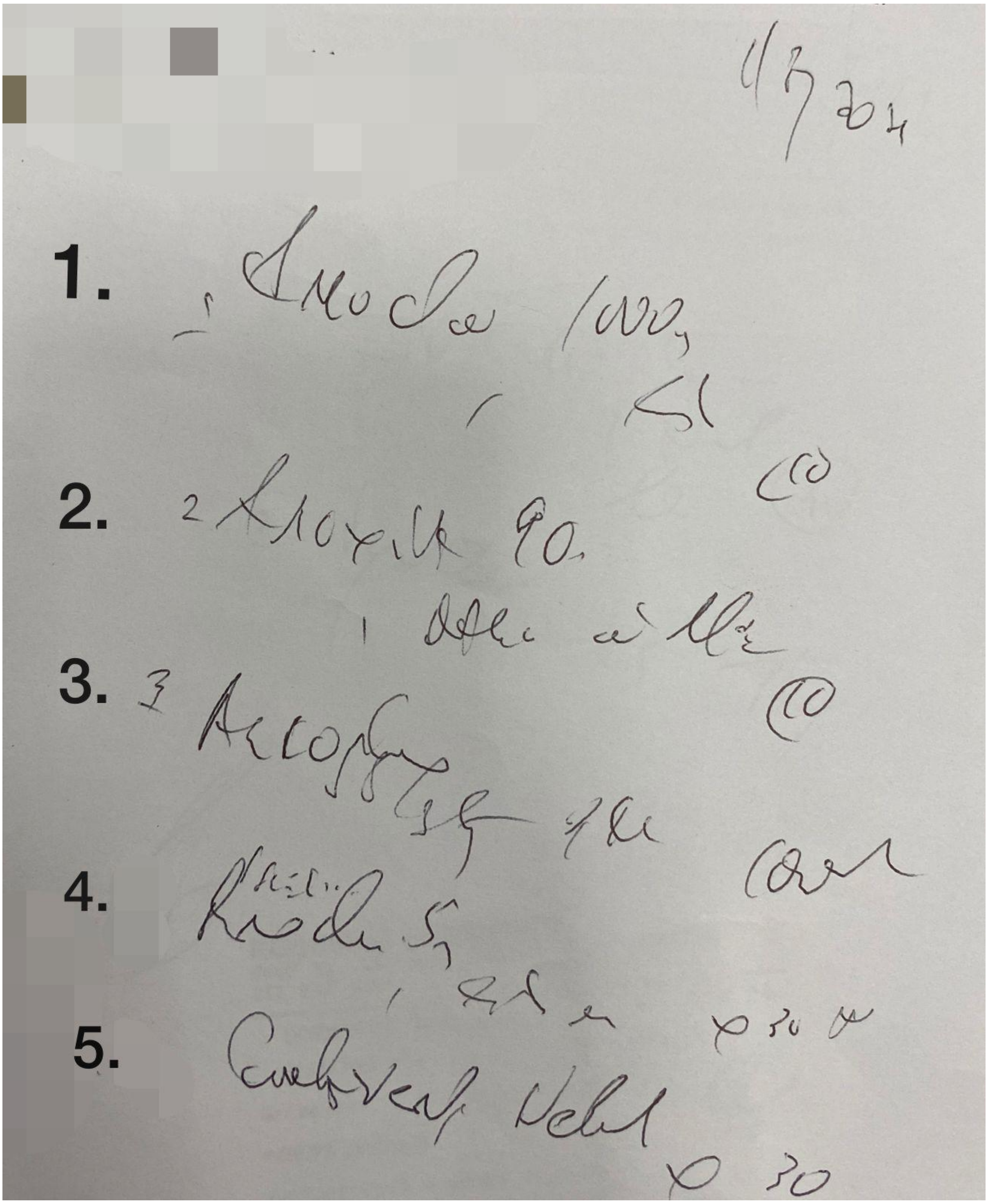

Prescription 3:

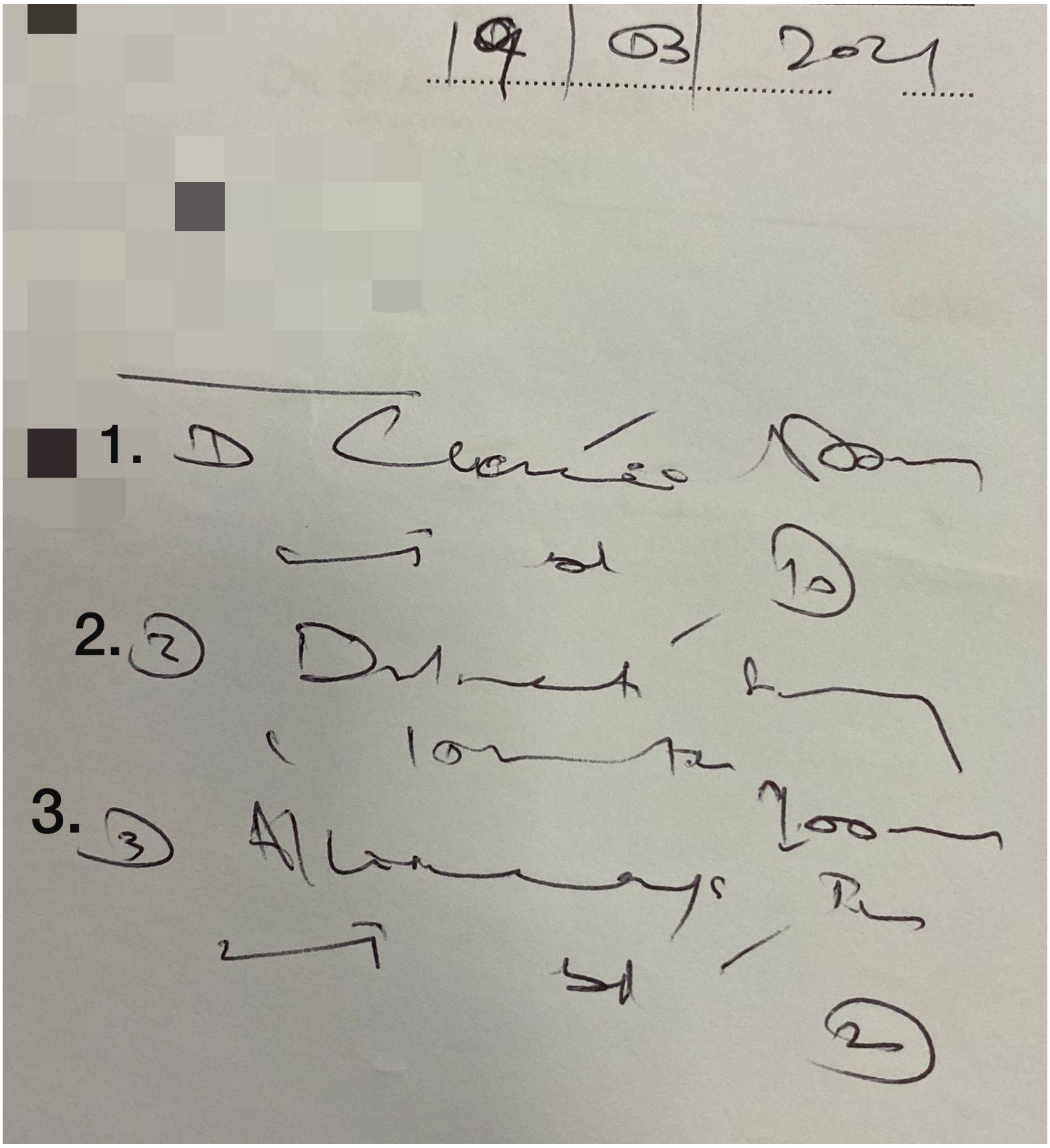

Prescription 4:

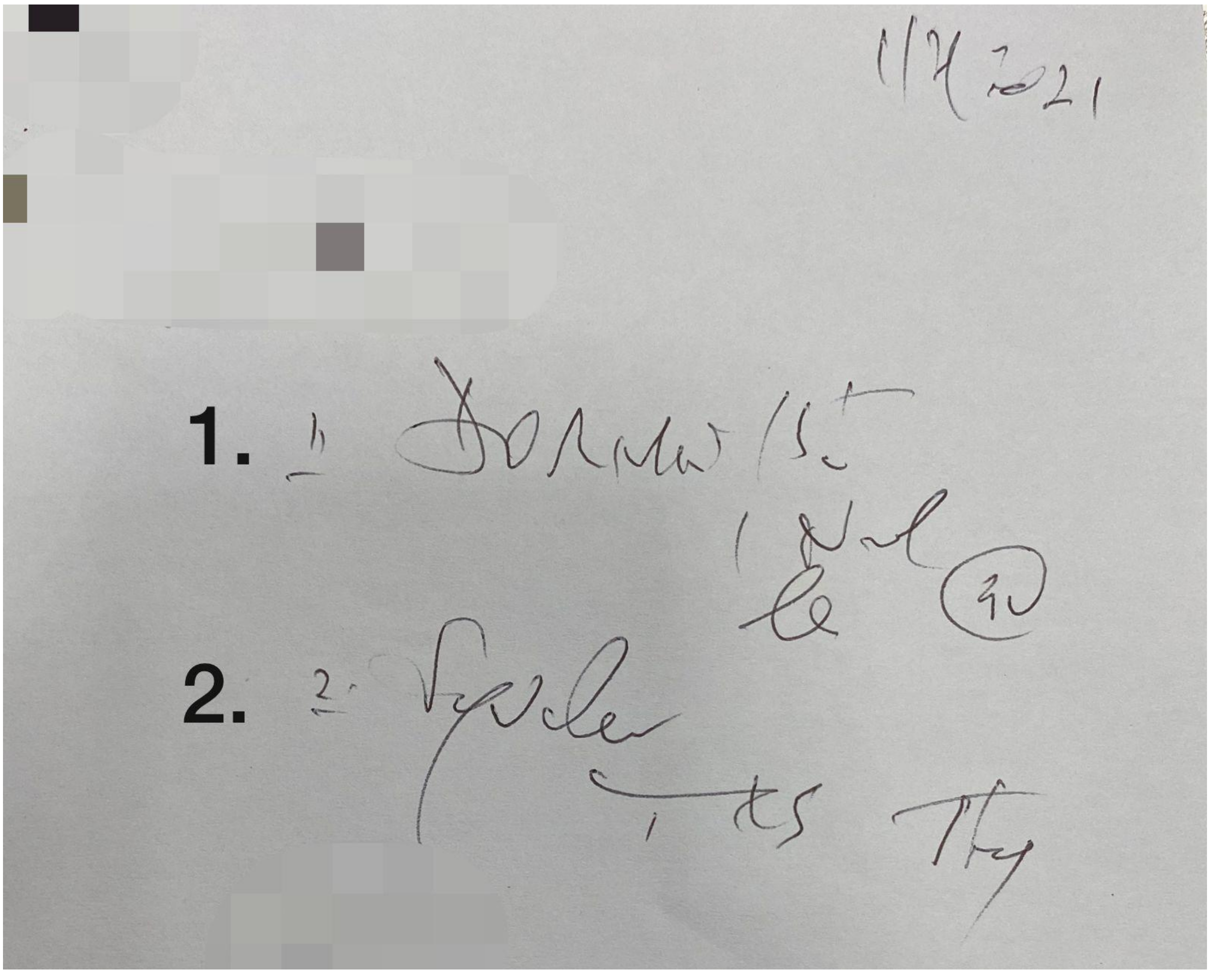

Prescription 5:

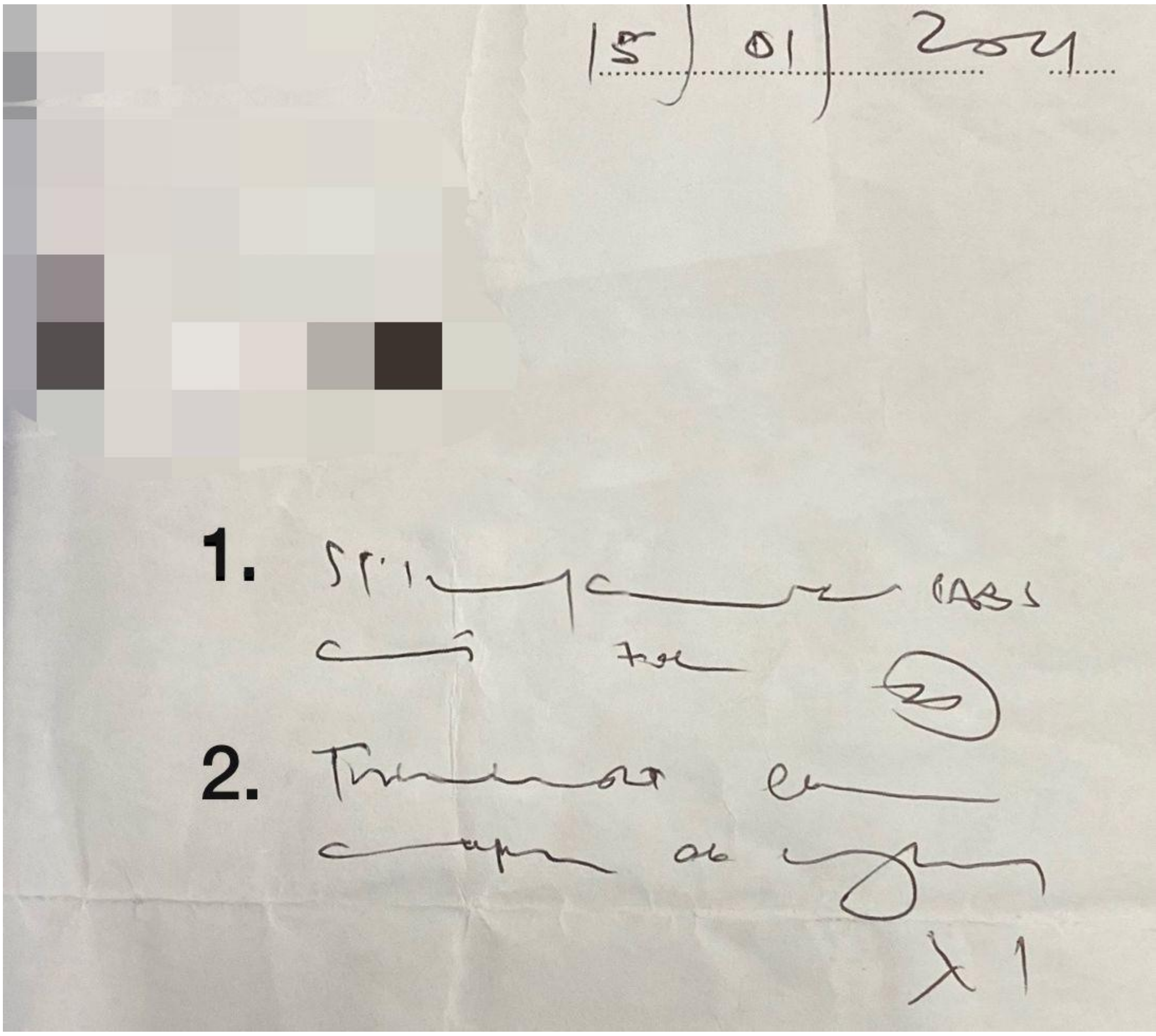

9. On a scale of 1-5 where:<br>1=Bad (impossible to read and identify medication)<br>2=Poor (illegible, words unclear and ambiguity exists)<br>3= Fair (some words illegible, some words legible, meaning unclear)<br>4=Good (most words legible)<br>5=Excellent (all words are clear)<br>**How legible would you rate this prescription?**	○ 1<br>○ 2<br>○ 3<br>○ 4<br>o 5		
10. Please type out the medication you think was prescribed.<br>medication 1:<br>medication 2:<br>medication 3:<br>medication 4:<br>medication 5:	**TEXTBOX**		
11. What was the date (e.g. day (calender number), month and year) the prescription was written?	**TEXTBOX**		
12. Can you easily read the abbreviations used?	○ Yes	○ No	○ Not Applicable
13. Type the abbreviation used:	**TEXTBOX**		
14. Can you easily read what quantity was prescribed?	○ Yes	○ No	

15. Please type out what quantity you think was prescribed	**TEXTBOX**	
16. Can you easily read the precise dosage?	○ Yes	○ No
17. Please type the precise dosage you think was prescribed.	**TEXTBOX**	
18. Can you easily read the dosage form?	○ Yes	○ No
19. Please type the dosage form below (oral, IV,IM etc.) below	**TEXTBOX**	
20. If you could not read this prescription would you call the doctor?	○ Yes	○ No
21. In general how would you rate the current system in place for dispensing prescriptions	○ Excellent<br>○ Good<br>○ Fair/Satisfactory<br>○ Poor<br>○ Bad	

Part 2: Survey with open-ended questions

**Question**	**Response (Type response in the textbox)**
1. How often do you receive an illegible prescription?	
2. Can you give a few words on the process you follow to decipher a sloppy prescription?	
3. Do you find this process time-consuming?	
4. Does receiving an illegible prescription affect service delivery in the pharmacy? If so, How?	
5. What are your biggest concerns/challenges around receiving a sloppy prescription?	
6. Have you ever faced patient frustration as the delivery of service gets delayed when handed a sloppy prescription?	
7. Would you like to share any fears or past experiences you have faced in dispensing sloppy prescriptions?	
8. What do you think are the consequences related to the dispensing of sloppy prescriptions?	
9. What are your thoughts on the current dispensing practices in place?	
10. What are your suggestions with regards to improving the accuracy of reading prescriptions?	
11. Any other comments or thoughts you would like to share?	
